# Ventriculo-atrial shunt with occlusion of the internal jugular vein: operative experience and surgical technique

**DOI:** 10.1186/s41016-023-00354-z

**Published:** 2024-01-12

**Authors:** Silvia Tatiana Quintero, Felipe Ramirez-Velandia, Andres Felipe Hortua Moreno, Lina Vera, Paula Rugeles, Rafael Augusto Azuero Gonzalez

**Affiliations:** 1 Pontificia Universidad Javeriana, Clinica Medilaser S.A.S., Clinica Chicamocha, Bucaramanga, Santander Colombia; 2 Neurology and Neurosurgery Research Group, Pontificia Universidad Javeriana, Bogotá́ D.C., Colombia; 3 Universidad Industrial de Santander, Clinica Chicamocha, Bucaramanga, Colombia; 4 Universidad Autónoma de Bucaramanga, Clinica Chicamocha, Bucaramanga, Colombia; 5 Clinica Chicamocha, Bucaramanga, Santander Colombia

**Keywords:** Hydrocephalus, Ventriculoatrial shunting, Internal jugular vein, Operative time, Complications, Shunt removal

## Abstract

**Background:**

Ventricular shunts are one of the most frequent techniques used for the management of hydrocephalus. The ventriculoperitoneal shunt (VPS) is the most commonly performed procedure, and the ventriculoatrial shunt (VAS) is the second option in most medical centers. The main objective of this study is to introduce and describe a surgical approach for VAS outlining our experience and comparing it with traditional shunting techniques.

**Methods:**

In this retrospective cohort comparison study, we included patients with hydrocephalus treated with a surgical procedure between January 2010 and February 2021 at a single academic institution. We categorized the procedures into two groups: patients with VPS and conventional VAS grouped together into the conventional technique (CT) group, and the second group was patients with whom we performed VAS with complete internal jugular vein occlusion (IJVOT). We compared the surgical time, postoperative complications, and occurrence of shunt failure among the groups by performing univariate analysis using the Fisher exact test.

**Results:**

Out of the 106 patients included in the analysis, IJVOT was performed in 66 patients, and CT in 40 patients. The median surgical time was 60 min (IQR 60–90) for IJVOT versus 100 min (IQR 60–120) for CT (*p* < 0.01). In the follow-up a month after the procedure, 83.3% of patients with IJVOT and 62.5% of patients with CT did not require shunt removal or shunt revision (*p* < 0.01). Shunt revision rates were 12.5% and 1.5% for CT while 1.5% and 2.5% for IJVOT at 1 and 6 months after the procedure.

**Conclusion:**

Our findings demonstrate that VAS with IJVOT is a safe method that exhibited shorter surgical times and outcomes comparable to CT. However, since the present study represents the first cohort evaluating IJVOT, it is imperative to conduct larger prospective studies, along with clinical trials, to fully explore and establish efficacy, long-term outcomes, and an in-depth comparison among shunting techniques.

## Background

Reports in the literature date back to about a century of efforts to find techniques and devices that adapt in the most physiological and least morbid way to the needs of cerebrospinal fluid (CSF) evacuation in the treatment of hydrocephalus [1, 2]. Some of the most used techniques are ventricular shunts and with them, there are several techniques in relation to the final site of the distal catheter. Sites of diversion include the peritoneum, right atrium, venous system of the neck, skull, pleura, gallbladder, and ureters [1, 3, 4]. Since the 1950s, with the advent of silicone catheters programmable valves, the ventriculoperitoneal shunt (VPS) emerged as the first alternative for managing all types of hydrocephalus, including the hydrocephalus in children and replacing the first described ventriculo-atrial shunting (VAS) [5–7].

However, studies have shown that shunt systems often require revision or reintervention, with only 25–33% of shunts reaching a 10-year lifespan without further intervention, and approximately 60–75% lasting 2 years without reintervention [3]. The outcomes appear to be slightly better for VPS; nonetheless, there are no randomized controlled trials comparing the various shunting techniques, making it difficult to identify the merits of one method over the other [8].

In the present paper, we describe our experience using a distinct approach that we have been using at our institution as the first alternative for managing hydrocephalus; ventriculo-atrial shunting with internal jugular vein occlusion. We present details about this technique, summarizing the critical points of the procedure, the catheter insertion, positioning, and fixation in the jugular vein, as well as the description of clinical results during the 1-year follow-up and comparison with conventional shunting techniques.

## Methods

### Study design

After obtaining IRB approval, we conducted a retrospective cohort comparison study, including all patients with hydrocephalus treated with a surgical procedure between January 2010 and February 2021 at a single academic institution (Clinica Chicamocha, Bucaramanga, Colombia).

### Exclusion criteria

**Fig. 1:**
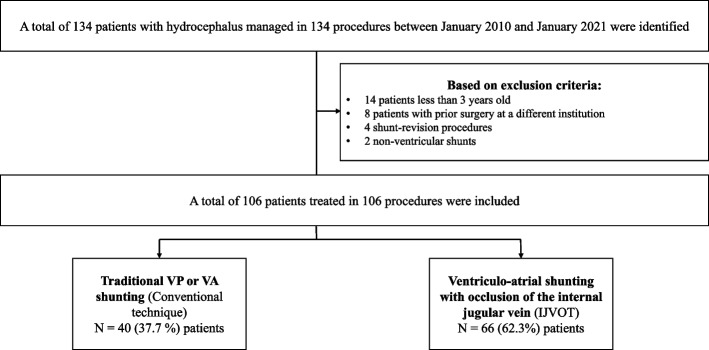
Flow chart of the exclusion process prior to analysis. *VP: ventriculoperitoneal; VA: ventriculoatrial; IJVOT: internal jugular vein occlusion technique

This study decided to exclude all patients with ventricular shunts performed under 3 years old (given that our VAS technique was not performed in patients under the age of three); patients with initial surgery in another institution, as it was not possible to establish the initial surgical technique; procedures that included shunt revision; and patients undergoing non-ventricular shunts (cysto-peritoneal shunt and subdural-peritoneal shunt). A detailed flowchart of the exclusion process is shown in Fig. 1.

### Variables and outcomes

We evaluated the results in two groups: patients with VPS and conventional VAS that conformed to the conventional technique (CT) group, and the second group were patients to whom we performed VAS with the complete internal jugular vein occlusion technique (IJVOT). The primary outcomes evaluated during the 1-year follow-up were shunt removal and shunt revision. Each electronic medical record was revised, and data were collected from the surgical description as well as follow-up appointments at the end of the first, sixth months, and 1 year after the date of the procedure to assess for complications, shunt removal, and shunt revision. The need for shunt revision was considered based on the physician’s discretion according to signs, symptoms, or imaging findings related to system malfunction.

### Statistical analysis

Procedures were categorized based on the shunting technique and compared through univariate analysis. Categorical variables were presented as proportions and analyzed using the *χ*2 test. Continuous variables, expressed as medians and dispersions with interquartile range (IQR) due to non-normal distribution, were compared using the Mann–Whitney *U* test. Fisher’s exact test was used to evaluate the association between each variable and the two outcomes of interest (shunt removal and shunt revision). A significance level of *P* = 0.05 was applied to all comparisons. Statistical analyses were conducted using STATA 17 (StataCorp, College Station, TX, USA).

### Ethics approval and consent to participate

The study was approved by the Human Subjects Committee in accordance with the Declaration of Helsinki of the World Medical Association and with Resolution 8430 (1993) of the Ministry of Health of Colombia. The patients/participants provided their written informed consent to participate in this study.

### Surgical technique

**Fig. 2:**
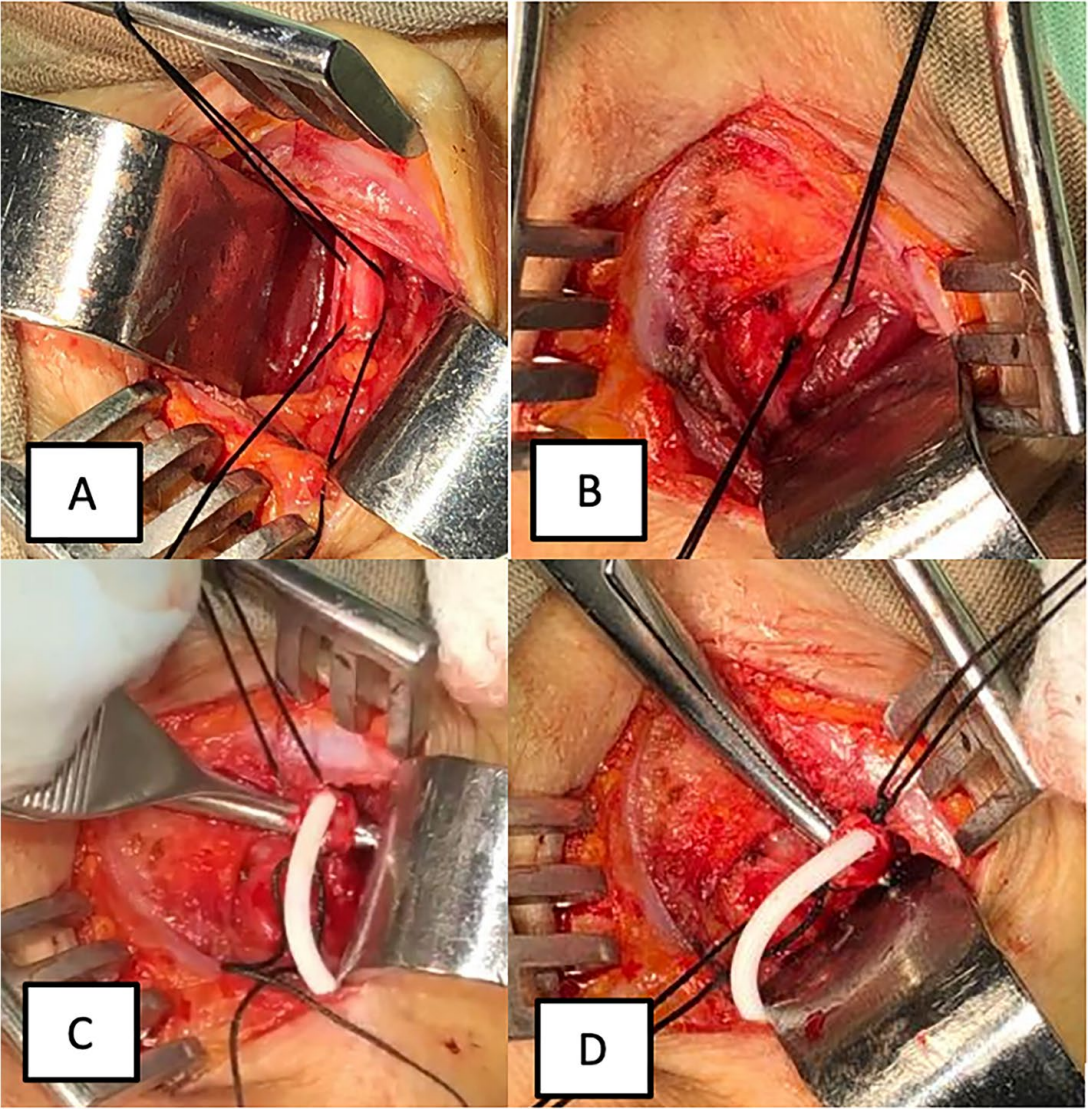
Critical steps in IJVOT. **A** Right internal jugular vein proximal and distal repair. The silk on the top represents the distal repair, and the one below represents the proximal repair. **B** Complete occlusion of the internal jugular vein proximal to the distal catheter entry (see the knot in the inferior silk). **C** Internal jugular vein incision, and insertion of the distal atrial catheter (catheter length is advanced 13 cm).** D** Distal catheter fixation to the vein

The patient is supine, under general anesthesia, and with a subscapular pillow to achieve mild neck extension. An oblique incision is made in the right anterior cervical region over the skin fold. Then, the platysma muscle is incised, followed by the separation of the sternocleidomastoid muscle laterally. Identification of the neck neurovascular bundle: After the internal jugular vein is separated from the carotid artery and the vagus nerve, a suture around the proximal and distal parts of the internal jugular vein is placed (Fig. 2A). The proximal catheter is positioned using the conventional shunting parameters, identifying the Kocher point, and directing it at an angle that is perpendicular to the intersection of lines drawn from the ipsilateral medial canthus and the ipsilateral external auditory meatus trying to position the ventricular catheter in the frontal horn of the lateral ventricle a level of Foramen of Monro [9]. Subsequently, we perform a complete occlusion of the internal jugular vein proximal to the distal catheter entry (Fig. 2B). Later, a venotomy is performed, and after the insertion of the distal atrial catheter in the internal jugular vein is realized, the distal catheter is advanced approximately 13 cm (Fig. 1C). Following this, we recommend catheter fixation to the vein with silk in the distal region (Fig. 1D). At the end, the revision of hemostasis and closure is done.

## Results

### Baseline patient characteristic

One hundred and six patients were included in our analysis; 66 patients in the IJVOT group (62.3%), and 40 in the CT group (37.7%). The median age was 50 years; the minimum age was 3 years old, and the maximum was 89 years old. There were slightly more females than males; communicating hydrocephalus was seen in 55 patients (51.8%), followed by obstructive hydrocephalus in 37 patients (34.6%), and lastly normal pressure hydrocephalus in 14 patients (13.1%). Causes varied, with the most common etiology identified as obstruction secondary to a posterior fossa tumor. The most common side of the procedure was the right and the most frequent type of valve used was the programmable Hakim valve, used in 83 patients (78.3%) (Table 1).

**Table 1:** Sociodemographic and general characteristics of both groups (IJVOT and CT)

	IJVOT *N* = 66	CT *N* = 40	Total *N* = 106	*p* value
Age^a^	49 (30–67)	53.5 (23.5–67)	50 (27–67)	0.69
Gender				
Males	30 (45%)	24 (60%)	54 (51%)	0.15
Females	36 (55%)	16 (40%)	52 (49%)	
Type of hydrocephalus				
Communicating	36 (55%)	19 (48%)	55 (52%)	0.28
Normal pressure hydrocephalus	6 (9%)	8 (20%)	14 (13%)	
Obstructive hydrocephalus	24 (36%)	13 (33%)	37 (35%)	
Etiology				
Subarachnoid hemorrhage	4 (10%)	11 (16.67%)	15 (14.15%)	0.45
Intracranial hemorrhage	2 (3.03%)	0 (0%)	2 (1.8%)	
Infectious	8 (12.12%)	6 (15%)	14 (13.21%)	
Trauma	3 (4.5%)	3 (7.5%)	6 (2.83%)	
Tumor	20 (30.3%)	12 (30%)	32 (30.18%)	
Congenital	5 (7.58%)	1 (2.5%)	6 (5.6%)	
Idiopathic	7 (10.6%)	7 (17.5%)	14 (13.2%)	
Other	17 (25.75%)	0 (0%)	17 (16.04%)	
Associated oncologic conditions				
Lung adenocarcinoma	2 (3%)	0 (0%)	2 (1.8%)	** < 0.01**
Breast adenocarcinoma	2 (3%)	0 (0%)	2 (1.8%)	
Shunt related variables				
Laterality of the shunt				
Right	59 (89%)	36 (90%)	95 (90%)	0.92
Left	7 (11%)	4 (10%)	11 (10%)	
Type of valve				
Hakim programmable valve	64 (96.96%)	19 (47.5%)	83 (78.3%)	** < 0.01**
Sophysa programmable valve	1 (1.52%)	0 (0)	1 (0.94%)	
Biomed with fixed pressure	1 (1.52%)	17 (42.5%)	18 (16.98%)	
Surgical time^a^				
	60 (60–90)	100 (60–120)	60 (60–120)	** < 0.01**

^a^Median (IQR), *p* value: Mann–Whitney *U* test

Bold numbers highlight *p* values with statistical significance

### Comparison of shunting technique

Univariate analysis comparing age, sex and type of hydrocephalus did not find a statistically significant difference between groups (*p* > 0.05) (Table 1). However, oncologic conditions and the placement of programmable Hakim valves were more common in the IJVOT group (*p* < 0.01). The median surgical time was 60 min for IJVOT and 100 min for conventional techniques; then, the surgical time was 40 min less in the group with IJVOT, which was statistically significant (*p* < 0.01).

### Outcomes

Only one surgical complication was registered in the study. This patient underwent IJVOT and developed a hematoma in the neck, which was identified early and required a new surgical procedure for revision and hemostasis. The bleeding was in the surgical bed but not from injury to the internal jugular vein. The hemostasis and closure were performed without further complications. No surgical complications were observed in the CT group.

In the follow-up 1 month after surgery, a shunt revision was required in 12.5% of patients in the CT group, compared to 1.5% of patients with IJVOT; and this difference was statistically significant (*p* = < 0.01). Most patients in the IJVOT group (83.3%) had the system unchanged at the end of the first month, compared to 62.5% in the CT group (*p* = < 0.01). Moreover, there was a trend of lower rates of shunt removal or revision in the IJVOT at 6 and 12 months, although this difference did not reach statistical significance (Table 2).

**Table 2:** Clinical outcomes during follow-up

	IJVOT	CT	Total	*p* value
	N (%)	N (%)	N (%)	
System unchanged				
1 month^b^	55 (83.3%)	25 (62.5%)	75 (70.57%)	** < 0.01**
6 months^b^	41 (62.1%)	22 (55%)	63 (64.94%)	0.15
12 months^c^	34 (51.5%)	18 (45%)	52 (60.47%)	0.20
Shunt revision				
1 month^a^	2 (1.5%)	5 (12.5%)	7 (6.60%)	** < 0.01**
6 months^b^	1 (2.5%)	1 (1.5%)	2 (2.06%)	0.15
Shunt removal				
1 month^a^	0 (0%)	2 (5%)	2 (1.89%)	** < 0.01**
6 months^b^	3 (4.55%)	2 (5%)	5 (5.15%)	0.15
12 months^c^	1 (2.5%)	1 (1.5%)	2 (2.32%)	0.20
Deaths	6 (9%)	10 (25%)	16 (15.09%)	0.21

^a^106 patients completed the 1-month follow-up, 66 for the IJVOT and 40 for the CT

^b^97 patients completed the 6-month follow-up, 64 for the IJVOT and 33 for the CT

^c^86 patients completed the 12-month follow-up, 58 for the IJVOT and 28 for the CT

Eighty-six patients completed the 1 year follow-up. Sixteen patients died during this period (as shown in Table 3); 10 of them were part of the CT group (25%), while the remaining 6 belonged to the IJVOT group (9%), but the difference did not reach statistical significance (*p* = 0.21). Most deaths occurred during the first month of follow-up and were secondary to hydrocephalus in association with life-threatening conditions. The causes of death were diverse and included autoimmune encephalitis (*n* = 1), aneurysmal subarachnoid hemorrhage (*n* = 3), post-infective hydrocephalus (*n* = 4), post-traumatic hydrocephalus (*n* = 1), posterior fossa tumor (*n* = 5), and supratentorial brain tumor (*n* = 1). Notably, only one patient with normal pressure hydrocephalus died; he presented with bilateral subdural hematomas 3 months after the shunt surgery. He required subdural hematoma drainage and succumbed one week after the procedure due to a sudden cardiorespiratory arrest. It is worth mentioning that this was the same patient who had experienced a neck hematoma following the shunt with IJVOT. Nevertheless, we could not establish a direct link between the procedure and the subsequent fatality.

**Table 3:** Deaths reported within 1 year after the surgical procedure

Diagnosis	IJVOT	CT	Total
	*n* (%)	*n* (%)	*n* (%)
Autoimmune encephalitis	0 (0)	1 (2.5%)	1 (0.9)
Aneurysmal subarachnoid hemorrhage	1 (1.5%)	2 (5)	3 (2.8)
Normal pressure hydrocephalus^a^	1 (1.5)	0 (0)	1 (0.9)
Post-infectious hydrocephalus	2 (3)	2 (5)	4 (3.7)
Post-traumatic hydrocephalus	0 (0)	1 (2.5)	1 (0.9)
Posterior fossa tumor	2 (3)	3 (7.5)	5 (4.7)
Supratentorial tumor	0 (0)	1 (2.5)	1 (0.9)

^a^Presented with bilateral subdural hematomas 3 months after IJVOT; he required subdural hematoma drainage and died one week after surgery due to sudden cardiorespiratory arrest

## Discussion

Despite the advances in shunting techniques and the high volume of VPS procedures, the failure rate remains high, and shunting is considered a temporary solution for the treatment of hydrocephalus. Up until this point, the ventriculoperitoneal shunt is the primary method of treatment of hydrocephalus. This is probably related to the fact that VAS needs to be checked as the child grows, and VPS are technically easier to monitor [10]. There is also a belief that VPS is technically simpler to perform and has fewer major complications [11, 12]. However, long-term outcomes have not shown significant differences among different CSF diversion techniques, and some studies have even reported higher rates of distal dysfunction with VPS [13]. Furthermore, survival rates do not differ significantly when comparing VPS with VAS, and the rates of infection and proximal failure have been reported to be lower in patients with VAS [3, 8].

Similar to our technique, studies have shown that VAS can be a convenient and safe alternative to VPS [8, 14, 15]. Some evidence suggests that VAS may provide higher flow rates due to the lower intra-atrial pressure compared to intra-abdominal pressure, leading to more consistent cerebrospinal fluid flow and potentially fewer malfunctions [16]. Currently, VAS are often used as a second-line treatment option after VPS failure [17], or in cases where increased intra-abdominal pressure is anticipated, such as during pregnancy [18], or in patients with ascites following VAS [19], and when there is a history of multiple abdominal surgeries.

In the literature, complications with VAS range from occlusion to bacteremia, thromboembolism, pneumothorax, hemothorax, arterial punction, hematoma, and arrhythmias [20]. However, only one complication was registered in our cohort. We think that to minimize the risk of these complications, training, proper technique, and adherence to sterile protocols are essential.

In the past, accessing the venous system for VAS placement often involved open dissection of the facial or external jugular vein, allowing for direct visualization of the distal catheter insertion into the venous system and directed towards the atrium [1]. This approach provided a clear view and minimized catheter placement difficulties, particularly in young children [21]. In our proposed technique, we suggest an open dissection of the internal jugular vein to guide the catheter to the atrium. The internal jugular vein is chosen due to its relatively consistent caliber and anatomy, which can facilitate the procedure, and the right side is often selected as a surgeon’s preference. Some studies have described percutaneous placement of the atrial catheter using fluoroscopic and ultrasound guidance [17, 22], but these technologies may not always be readily available, especially in underdeveloped countries or resource-limited settings.

It is important to consider the hemodynamic changes that may occur after occlusion of the internal jugular vein (IJV). In the past, there was concern that unilateral occlusion of the IJV could potentially increase intracranial pressure (ICP) and decrease CSF absorption [23]. However, our understanding of this concept has evolved. This is due to the compensatory mechanisms of the contralateral jugular drainage and the presence of non-jugular venous drainage pathways, such as the vertebral plexus and pterygopalatine plexus [24]. When one IJV is obstructed, the other vein can compensate by forming collateral vessels, and the non-jugular venous system can also contribute to venous drainage. Case reports in patients with metastatic head and neck cancers have shown that bilateral sacrifice of jugular veins can be tolerated without significant ICP elevation [25]. However, it is important to note that in some cases, patients may develop papilledema after unilateral jugular vein thrombosis, but this is typically due to associated cerebral venous sinus thrombosis [26] or a hypoplastic contralateral transverse sinus [27]. Therefore, an acute increase in ICP after occlusion of the IJV is unlikely, unless there is an associated venous abnormality or shunt malfunction. The shunting system itself provides a compensatory mechanism to divert flow in cases where ICP increases. This allows for appropriate drainage and regulation of CSF dynamics. Nonetheless, individual patient factors and pre-existing venous abnormalities should be taken into consideration when planning and performing IJV occlusion for shunt placement.

Our research findings demonstrate that VAS with IJVOT is an easy and safe technique, which offers several advantages over conventional techniques. Notably, this approach requires less surgical time and yields similar long-term results. The significance of shortening surgical time is underscored by well-documented evidence that it may accelerate postoperative recovery and rehab after surgery [28]. Additionally, the cervical approach utilized in this technique is accessible in any healthcare institution worldwide, including developing countries like Colombia. As a result, we have implemented this described technique as the primary alternative for ventricular shunting in adult patients with hydrocephalus, achieving comparable outcomes to those of conventional techniques.

Despite the significant findings of our study, it is important to acknowledge several limitations. Firstly, our sample consisted of 106 patients with hydrocephalus at a single center in Colombia limiting generalizability to other clinical contexts. Secondly, the retrospective nature of this study introduces inherent bias when establishing comparisons. Thirdly, we did not provide a detailed comparison of outcomes for each type of conventional shunting technique, as we chose to group both the VPS and the conventional ventriculoatrial VAS. Fourthly, the follow-up period was limited to 1 year, potentially missing out on long-term system dysfunction and the need for removal, which may occur over a longer timeframe. Additionally, we did not measure intracranial pressure, cerebral oxygen saturation (rSO2), and bispectral index (BIS), which are important parameters to consider after occluding the internal jugular vein. Therefore, future studies should incorporate these measurements and employ a longer follow-up duration to provide more comprehensive insights into the benefits of this technique.

## Conclusion

The VAS with IJVOT presents a potential option for ventricular shunting in adult patients with hydrocephalus. Although our study contributes valuable insights, it is crucial to recognize that further research including the development of prospective cohorts, and clinical trials is necessary to fully explore and establish the efficacy, and comparability of this technique.

## Data Availability

The datasets used and/or analyzed during the current study are available from the corresponding author on reasonable request.

## Abbreviations

VP: Ventriculoperitoneal
VPS: Ventriculoperitoneal shunt
VA: Ventriculoatrial
VAS: Ventriculoatrial shunt
IJVOT: Internal jugular vein occlusion technique
CT: Conventional technique

## References

[1] Tomei KL (2017). The evolution of cerebrospinal fluid shunts: advances in technology and technique. Pediatr Neurosurg.

[2] Symss NP, Oi S (2015). Is there an ideal shunt? a panoramic view of 110 years in CSF diversions and shunt systems used for the treatment of hydrocephalus: from historical events to current trends. Child’s Nerv Syst ChNS Off J Int Soc Pediatr Neurosurg.

[3] Iglesias S, Ros B, Martín Á, Carrasco A, Segura M, Delgado A (2016). Surgical outcome of the shunt: 15-year experience in a single institution. Child’s Nerv Syst ChNS Off J Int Soc Pediatr Neurosurg.

[4] Pillai A, Mathew G, Nachimuthu S, Kalavampara SV. Ventriculo-ureteral shunt insertion using percutaneous nephrostomy: a novel minimally invasive option in a patient with chronic hydrocephalus complicated by multiple distal ventriculoperitoneal shunt failures. J Neurosurg. 2017;1–5. 10.3171/2016.8.JNS16342.test 28306420

[5] Ignelzi RJ, Kirsch WM (1975). Follow-up analysis of ventriculoperitoneal and ventriculoatrial shunts for hydrocephalus. J Neurosurg.

[6] Keucher TR, Mealey JJ (1979). Long-term results after ventriculoatrial and ventriculoperitoneal shunting for infantile hydrocephalus. J Neurosurg.

[7] Lam CH, Villemure JG (1997). Comparison between ventriculoatrial and ventriculoperitoneal shunting in the adult population. Br J Neurosurg.

[8] Rymarczuk GN, Keating RF, Coughlin DJ, Felbaum D, Myseros JS, Oluigbo C (2020). A comparison of ventriculoperitoneal and ventriculoatrial shunts in a population of 544 consecutive pediatric patients. Neurosurgery.

[9] Morone PJ, Dewan MC, Zuckerman SL, Tubbs RS, Singer RJ (2020). Craniometrics and ventricular access: a review of Kocher’s, Kaufman’s, Paine’s, Menovksy’s, Tubbs’, Keen’s, Frazier’s, Dandy’s, and Sanchez’s points. Oper Neurosurg (Hagerstown, Md).

[10] Mansoor N, Solheim O, Fredriksli OA, Gulati S (2021). Shunt complications and revisions in children: a retrospective single institution study. Brain Behav.

[11] Badhiwala JH, Kulkarni A V. Ventricular shunting procedures. Youmans and winn neurological surgery. 201; 2023. 1615–1629. 8th ed. Elsevier. [cited 2023 Feb 4]. Available from: https://www.clinicalkey.com/#!/content/book/3-s2.0-B978032328782100201X

[12] Borgbjerg BM, Gjerris F, Albeck MJ, Hauerberg J, Børgesen SV (1998). A comparison between ventriculo-peritoneal and ventriculo-atrial cerebrospinal fluid shunts in relation to rate of revision and durability. Acta Neurochir (Wien)..

[13] Giordan E, Palandri G, Lanzino G, Murad MH, Elder BD. Outcomes and complications of different surgical treatments for idiopathic normal pressure hydrocephalus: a systematic review and meta-analysis. J Neurosurg. 2018:1–13. 10.3171/2018.5.JNS1875. 10.3171/2018.5.JNS1875 30497150

[14] Mazza C, Pasqualin A, Da Pian R (1980). Results of treatment with ventriculoatrial and ventriculoperitoneal shunt in infantile nontumoral hydrocephalus. Childs Brain.

[15] Hung AL, Vivas-Buitrago T, Adam A, Lu J, Robison J, Elder BD (2017). Ventriculoatrial versus ventriculoperitoneal shunt complications in idiopathic normal pressure hydrocephalus. Clin Neurol Neurosurg.

[16] Suyama D (2022). Indication and technique of ventriculo: atrial shunt for Normal pressure hydrocephalus. No Shinkei Geka.

[17] Zhang J, Qu C, Wang Z, Wang C, Ding X, Pan S (2009). Improved ventriculoatrial shunt for cerebrospinal fluid diversion after multiple ventriculoperitoneal shunt failures. Surg Neurol..

[18] Murakami M, Morine M, Iwasa T, Takahashi Y, Miyamoto T, Hon PK (2010). Management of maternal hydrocephalus requires replacement of ventriculoperitoneal shunt with ventriculoatrial shunt: a case report. Arch Gynecol Obstet.

[19] Gil Z, Beni-Adani L, Siomin V, Nagar H, Dvir R, Constantini S (2001). Ascites following ventriculoperitoneal shunting in children with chiasmatic-hypothalamic glioma. Child’s Nerv Syst ChNS Off J Int Soc Pediatr Neurosurg.

[20] Hanak BW, Bonow RH, Harris CA, Browd SR (2017). Cerebrospinal fluid shunting complications in children. Pediatr Neurosurg.

[21] Erdogan H, Altun A, Kuruoglu E, Kaya AH, Dagcinar A. Difficulties of Distal Catheter Insertion of Ventriculoatrial Shunting in Infants and Little Children. Turk Neurosurg. 2018;28(4):66366. 10.5137/1019-5149.JTN.21304-17.2. 10.5137/1019-5149.JTN.21304-17.2 29091252

[22] Britz GW, Avellino AM, Schaller R, Loeser JD (1998). Percutaneous placement of ventriculoatrial shunts in the pediatric population. Pediatr Neurosurg.

[23] Kawajiri H, Furuse M, Namba R, Kotani J, Oka T (1983). Effect of internal jugular vein ligation on resorption of cerebrospinal fluid. J Maxillofac Surg.

[24] Kopelman M, Glik A, Greenberg S, Shelef I (2013). Intracranial nonjugular venous pathways: a possible compensatory drainage mechanism. AJNR Am J Neuroradiol.

[25] Ensari S, Kaptanoğlu E, Tun K, Gün T, Beşkonakli E, Celikkanat S (2008). Venous outflow of the brain after bilateral complete jugular ligation. Turk Neurosurg.

[26] Karaman E, Saritzali G, Cansiz H (2009). A case of increased intracranial pressure after unilateral modified radical neck dissection. Am J Otolaryngol.

[27] Scerrati A, Menegatti E, Zamboni M, Malagoni AM, Tessari M, Galeotti R, Zamboni P. Internal Jugular Vein Thrombosis: Etiology, Symptomatology, Diagnosis and Current Treatment. Diagnostics (Basel). 2021;11(2):378. 10.3390/diagnostics11020378. 10.3390/diagnostics11020378 PMC7926529 33672254

[28] Nigim F, Thomas AJ, Papavassiliou E, Schneider BE, Critchlow JF, Chen CC (2014). Ventriculoperitoneal shunting: laparoscopically assisted versus conventional open surgical approaches. Asian J Neurosurg.

